# An apicomplexan bromodomain protein, TgBDP1, associates with diverse epigenetic factors to regulate essential transcriptional processes in *Toxoplasma gondii*

**DOI:** 10.1128/mbio.03573-22

**Published:** 2023-06-23

**Authors:** Krista Fleck, Seth McNutt, Feixia Chu, Victoria Jeffers

**Affiliations:** 1 Molecular, Cellular, and Biomedical Sciences, University of New Hampshire, Durham, New Hampshire, USA; Stanford University, Stanford, California, USA

**Keywords:** *Toxoplasma gondii*, histone acetylation, chromatin, transcriptional regulation, molecular biology

## Abstract

The protozoan pathogen *Toxoplasma gondii* relies on tight regulation of gene expression to invade and establish infection in its host. The divergent gene regulatory mechanisms of *Toxoplasma* and related apicomplexan pathogens rely heavily on regulators of chromatin structure and histone modifications. The important contribution of histone acetylation for *Toxoplasma* in both acute and chronic infection has been demonstrated, where histone acetylation increases at active gene loci. However, the direct consequences of specific histone acetylation marks and the chromatin pathway that influences transcriptional regulation in response to the modification are unclear. As a reader of lysine acetylation, the bromodomain serves as a mediator between the acetylated histone and transcriptional regulators. Here we show that the bromodomain protein, TgBDP1, which is conserved among Apicomplexa and within the Alveolata superphylum, is essential for *Toxoplasma* asexual proliferation. Using cleavage under targets and tagmentation, we demonstrate that TgBDP1 is recruited to transcriptional start sites of a large proportion of parasite genes. Transcriptional profiling during TgBDP1 knockdown revealed that loss of TgBDP1 leads to major dysregulation of gene expression, implying multiple roles for TgBDP1 in both gene activation and repression. This is supported by interactome analysis of TgBDP1 demonstrating that TgBDP1 forms a core complex with two other bromodomain proteins and an ApiAP2 factor. This core complex appears to interact with other epigenetic factors such as nucleosome remodeling complexes. We conclude that TgBDP1 interacts with diverse epigenetic regulators to exert opposing influences on gene expression in the *Toxoplasma* tachyzoite.

**IMPORTANCE:**

Histone acetylation is critical for proper regulation of gene expression in the single-celled eukaryotic pathogen *Toxoplasma gondii*. Bromodomain proteins are “readers” of histone acetylation and may link the modified chromatin to transcription factors. Here, we show that the bromodomain protein TgBDP1 is essential for parasite survival and that loss of TgBDP1 results in global dysregulation of gene expression. TgBDP1 is recruited to the promoter region of a large proportion of parasite genes, forms a core complex with two other bromodomain proteins, and interacts with different transcriptional regulatory complexes. We conclude that TgBDP1 is a key factor for sensing specific histone modifications to influence multiple facets of transcriptional regulation in *Toxoplasma gondii*.

## INTRODUCTION

The protozoan *Toxoplasma gondii* is a ubiquitous parasite, infecting a third of the world’s human population and vast numbers of livestock and wildlife in sensitive ecosystems. *Toxoplasma* is a member of the phylum Apicomplexa, which contains many important pathogens such as the intestinal parasite *Cryptosporidium* and *Plasmodium* the causative agent of malaria. Infections by this group of intracellular parasites are notoriously difficult to treat or prevent due to their conserved eukaryotic cellular functions and their complex life cycles.

In addition to cell growth and maintenance, rapid molecular changes are required for *Toxoplasma* to transition between life cycle stages to support the establishment and persistence of infection. Regulation of chromatin structure to support appropriate gene expression is vital; however, these critical mechanisms in *Toxoplasma* are not fully understood (1, 2). The addition and removal of post-translational modifications on histones and the importance of this dynamic in regulating transcription have become evident (1, 3). Acetylation of lysine residues on histones plays a particularly important role in transcriptional activation (4 - 10). Similarly to observations in other eukaryotes, abolishing the function of *Toxoplasma* lysine acetyltransferases, or lysine deacetylases, the enzymes responsible for the addition or removal of acetyl groups on histones, perturbs gene expression and disrupts parasite proliferation and life cycle progression (9, 11 - 13). These enzymes have been a focus of investigation for drug discovery; however, a key player in the acetylation network, the bromodomain, is relatively understudied. The bromodomain consists of an approximately 110 amino acid sequence that forms an alpha helical bundle with a hydrophobic pocket that “reads” (recognizes and binds) the acetyl group on a lysine residue (14). Proteins containing bromodomains may perform multiple functions once bound to their intended targets, largely recruiting and interacting with other complexes to modify chromatin and regulate transcription. Due to their diverse and critical function, and their amenability to small-molecule inhibitors, bromodomains have become promising therapeutic targets in humans as treatments for cancer and immune and metabolic disorders (15).

A handful of bromodomain-containing proteins have recently been identified as key regulators of transcription and potential drug targets in Apicomplexans. In *Plasmodium falciparum,* a complex consisting of bromodomain proteins PfBDP1, PfBDP2, and PfBDP7 contributes to the expression of invasion factors (16, 17) and, in parallel, functions as a repressor complex to maintain mutually exclusive expression of variant surface antigens (VSAs) (18). The bromodomain of a GCN5 homologue in *Toxoplasma* (TgGCN5b) was found to be important for parasite growth and a target of the bromodomain inhibitor L-Moses (11). Another bromodomain inhibitor, IBET-151, was also reported to inhibit *Toxoplasma* tachyzoite proliferation (19). Twelve predicted proteins with conserved bromodomains have been identified in the *Toxoplasma* genome, but six of these (TgBDP1-6) are unique to early-branching eukaryotes (20, 21). While these parasite-specific bromodomain proteins have excellent therapeutic potential, they have yet to be studied in *Toxoplasma* and their functions are unknown.

In the present study, we sought to determine the role of the parasite-specific bromodomain protein TgBDP1 in *Toxoplasma* tachyzoites and validate its potential as a therapeutic target. Through sequence and structure analysis, we confirmed TgBDP1 has a conserved bromodomain with homologues displaying a similar domain architecture only present in early-branching alveolates. We generated a tetracycline-regulatable *tgbdp1* knockdown line and show that TgBDP1 is essential for progression through the parasite lytic cycle. We adapted and performed the cleavage under targets and tagmentation (CUT&Tag) technique for the first time in a protozoan, revealing that TgBDP1 is recruited to transcriptional start sites (TSS) and performed co-immunoprecipitations (coIPs) to identify interacting proteins. Further supporting its role in transcription, knockdown resulted in substantial disruption to parasite gene expression. We conclude that TgBDP1 is an essential component of several key transcriptional regulatory complexes.

## RESULTS

### TgBDP1 is a bromodomain-containing protein conserved in apicomplexans

**Fig 1:**
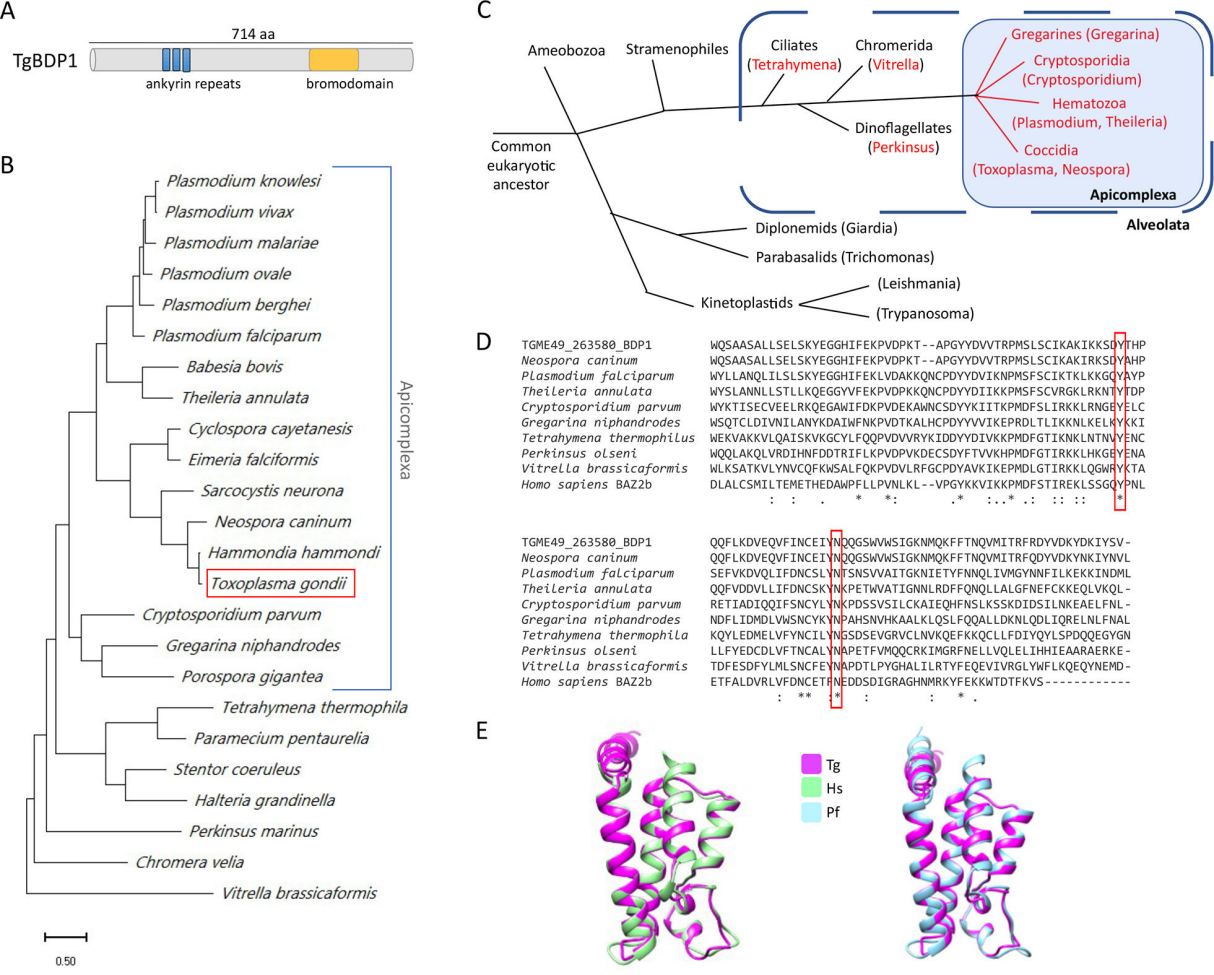
TgBDP1 is a bromodomain-containing protein that is conserved among alveolates. (**A**) Depiction of TgBDP1 protein size and domain architecture. (**B**) Phylogenetic tree of TgBDP1 protein homologues drawn to scale, with branch lengths measured in the number of substitutions per site. (**C**) Evolutionary tree with genera containing predicted TgBDP1 homologues in red. Apicomplexans are in blue-shaded box, and blue-dotted line encompasses alveolates. Branch lengths are not to scale. (**D**) Multiple alignment of bromodomain amino acid sequences from representative species, with TgBDP1 denoted as TGME49_263580 BDP1. The highly conserved tyrosine (Y) and asparagine (N) residues required for binding acetylated lysines are boxed in red. (**E**) The predicted structure of the TgBDP1 bromodomain (pink) overlaid with the *Homo sapiens* B2AZB bromodomain (green, PDB:5DYU) and *P. falciparum* PfBDP1 bromodomain (blue, PDB:7M97).

The *Toxoplasma* genome encodes 12 bromodomain-containing proteins, 6 of which (named TgBDP1-TgBDP6) have no homologues in mammals, plants, or fungi (20, 21). TgBDP1 (TGME49_263580) is predicted to be a 76-kDa protein containing a single bromodomain and a series of three ankyrin repeats (Fig. 1A). This domain architecture is only found in proteins from a subset of alveolates. BLAST analyses and conserved domain searches identified homologues in all Apicomplexan species examined but only a handful of other alveolates (Fig. 1B and C). The bromodomain is highly conserved and contains the characteristic tyrosine (Y) and asparagine (N) residues necessary for domain binding to acetylated lysines (Fig. 1D) (22). The structure of the bromodomain of TgBDP1 was modeled using I-TASSER and compared with experimentally determined bromodomain structures (23). The predicted structure of TgBDP1 is highly similar to that of the bromodomain from the human protein BAZ2B, forming an alpha helical bundle and hydrophobic binding pocket in which the residues required for coordination of the target acetylated lysine are present and appropriately positioned (Fig. 1E). The crystal structure of the bromodomain of *P. falciparum* PfBDP1 has recently been determined (PDB: 7M97) (24) and closely matches that of the predicted structure of the bromodomain in TgBDP1. The conserved sequence and structure of the predicted TgBDP1 bromodomain suggest that TgBDP1 is a functional acetyl-lysine reader.

### TgBDP1 has an mRNA isoform

**Fig 2:**
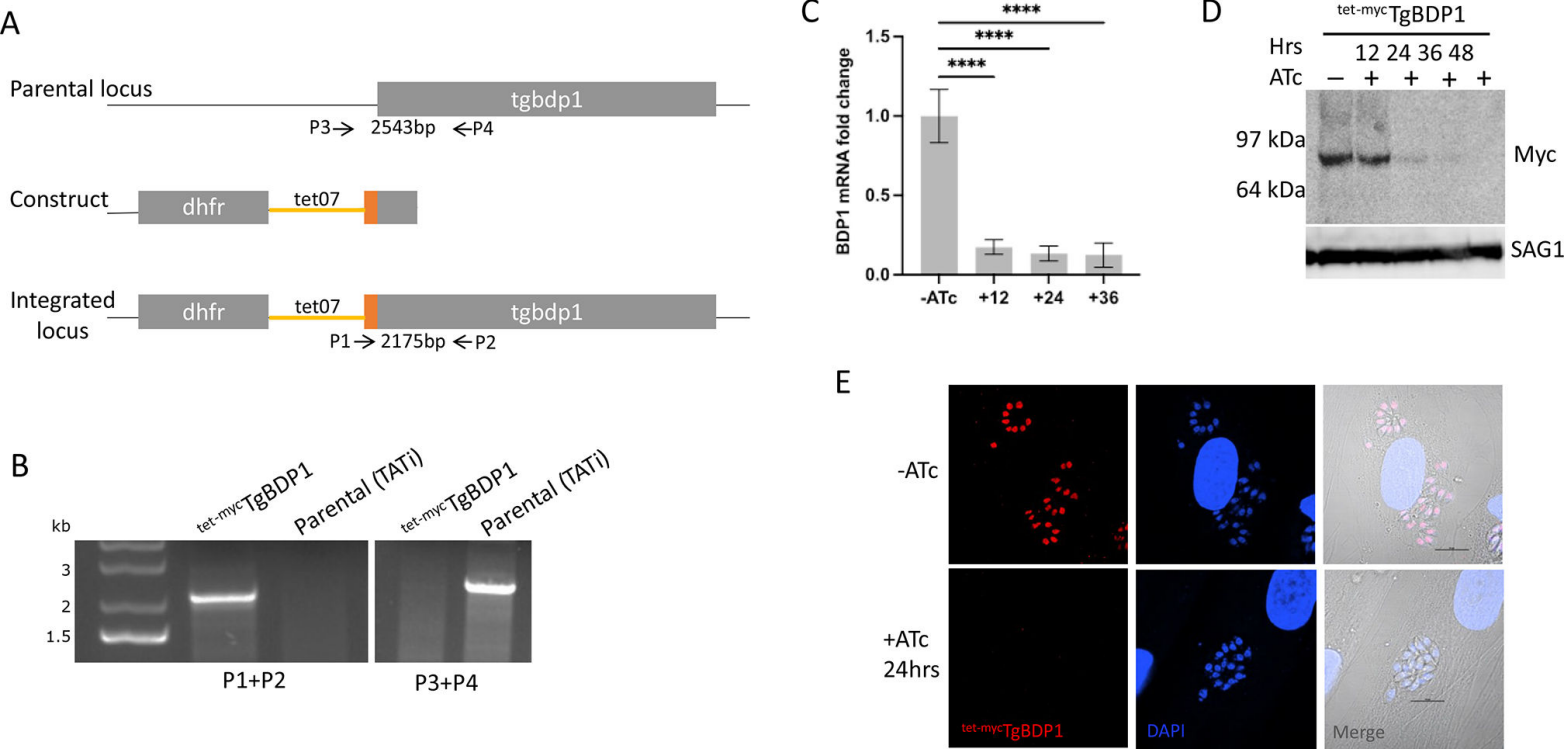
Generation of a *tgbdp1* inducible knockdown. (**A**) Strategy for *tgbdp1* promoter replacement with a tetracycline-regulatable promoter and insertion of an N-terminal myc tag (orange). The *dhfr* gene was inserted for selection of transgenic parasites with pyrimethamine resistance. Primers used to confirm integration are included. (**B**) PCRs confirming correct genomic modification. Primers P1 and P2 amplify a 2,175-bp fragment only present in the transgenic line (^tet-myc^TgBDP1), and primers P3 and P4 amplify a 2,543-bp fragment only in the parental (TATi) genome. (**C**) RT-qPCR of *tgbdp1* mRNA levels normalized to the −ATc sample, *n* = 3, **** = *P*-value < 0.0001. (**D**) Western blotting of ^tet-myc^TgBDP1 lysates from parasites cultured −ATc and +ATc for 12, 24, 36, and 48 h. TgSAG1 was included as a loading control. (**E**) IFA of ^tet-myc^TgBDP1 parasites cultured 24 h −ATc and +ATc.

The predicted protein sequence of TgBDP1 contains 714 amino acids. However, intron predictions based on available RNA-sequencing data in the *Toxoplasma* genome database ToxoDB (25) predicted two possible sizes for the first exon (Fig. S1A). The transcript with the longer exon matches the predicted mRNA sequence for *tgbdp1*, and the transcript containing the shorter exon produces an isoform with 63 fewer nucleotides, equivalent to a loss of 21 amino acids (Fig. S1B). High-resolution nanopore sequencing of *Toxoplasma* mRNAs conducted by Lee et al. also detected the two isoforms (Fig. S1A) (26). To confirm these findings, we amplified and sequenced *tgbdp1* cDNA. Half of the clones sequenced (3/6) contained the shorter isoform, which we named *tgbdp1a* (Fig. S1C). Additionally, our RNA-sequencing data from a separate line of experiments (described later) showed *tgbdp1* peak variations in all three replicates that would be consistent with a mixed population of the two mRNA isoforms (Fig. S1D). The TgBDP1 and TgBDP1a proteins are predicted to be 76 and 74.5 kDa, respectively. However, this small difference in size cannot be distinguished by Western blotting of N-terminally or C-terminally tagged TgBDP1 (Fig. 2D and 4A). The remaining experiments outlined in this study are focused on TgBDP1 that was modified at the endogenous genomic locus, and thus TgBDP1 isoforms would presumably be processed and function as normal. Additional studies will be needed to determine if these two isoforms have distinct functions.

### Generation of TgBDP1 inducible knockdown

A genome-wide CRISPR screen to evaluate the essentiality of genes in *Toxoplasma* tachyzoites reported a low fitness score for *tgbdp1*, indicating that this gene could be essential (27). To determine the role of TgBDP1, we generated a transgenic, inducible knockdown line ^tet-myc^TgBDP1 in which the endogenous *tgbdp1* promoter is replaced with a hybrid of the *Toxoplasma sag4* promoter and the tetracycline-responsive promoter, and a sequence encoding a triple myc tag is inserted at the 5′ end of the gene (Fig. 2A). Correct genomic integration was confirmed by PCR (Fig. 2B), and nuclear localization of TgBDP1 protein was determined by immunofluorescence (IFA) (Fig. 2E). Treatment with anhydrotetracycline (ATc) to abolish transcription of *tgbdp1* results in a significant reduction of *tgbdp1* mRNA levels (Fig. 2C). TgBDP1 protein also decreased over time as seen by Western blotting and IFA, falling below detectable levels by 36 h (Fig. 2D and E).

### TgBDP1 is essential for the tachyzoite lytic cycle

**Fig 3:**
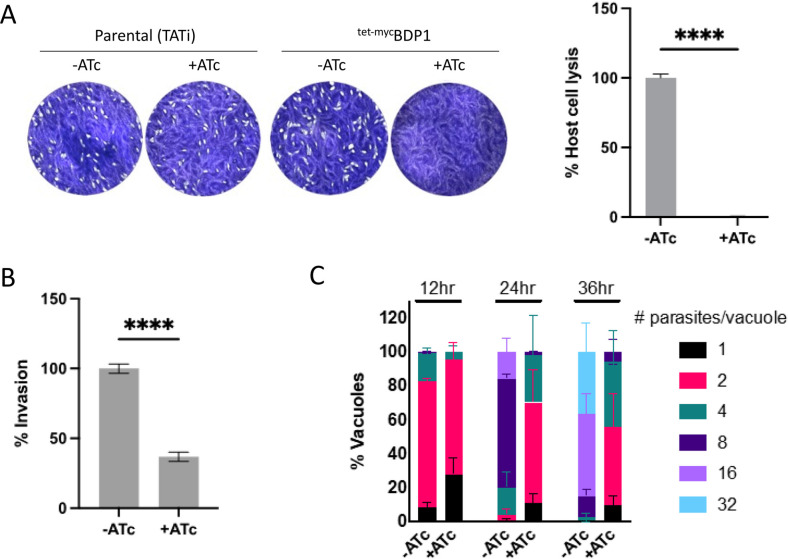
Loss of TgBDP1 causes significant defects in host cell invasion and parasite replication. (**A**) Plaque assays were conducted with the ^tet-myc^TgBDP1 and parental (TATi) parasite lines −ATc and +ATc. Images are representative from three independent experiments after 6 d of growth. The area lysed was calculated as a percentage of −ATc. (**B**) Invasion assays were performed by counting the number of parasites that invaded host cells and calculated as a percentage of −ATc. (**C**) Doubling assays were performed by counting the number of parasites per vacuole for 100 vacuoles at 12, 24, and 36 h after inoculation and plotted as a percentage of the total number of vacuoles. All experiments were done in triplicate, and unpaired *t*-tests performed for plaque and invasion assays, *n* = 3, **** = *P*-value < 0.0001.

Plaque assays were performed to evaluate parasite proliferation in the absence of TgBDP1. Tachyzoites of ^tet-myc^TgBDP1 did not form plaques in the presence of ATc over 6 d, unlike the parental parasite line (TATi) that grew normally and formed plaques in the presence and absence of ATc (Fig. 3A). To determine the precise point in the parasite’s lytic cycle that is inhibited, we tested the ability of parasites to invade and replicate within host cells. Parasites exposed to ATc for 36 h displayed a threefold reduction in their ability to invade cells (Fig. 3B). Defects in replication were seen as early as 12 h post-invasion in the presence of ATc (Fig. 3C). By 36 h, when viewed by light microscopy, parasites were deformed and completely stalled in their replication. No defects in replication were observed when the parental line (TATi) was exposed to ATc (Fig. S2). These results demonstrate that loss of TgBDP1 severely impedes tachyzoite host cell invasion and replication, and TgBDP1 is essential for parasite proliferation.

### TgBDP1 functions as part of a parasite-specific core complex

**Fig 4:**
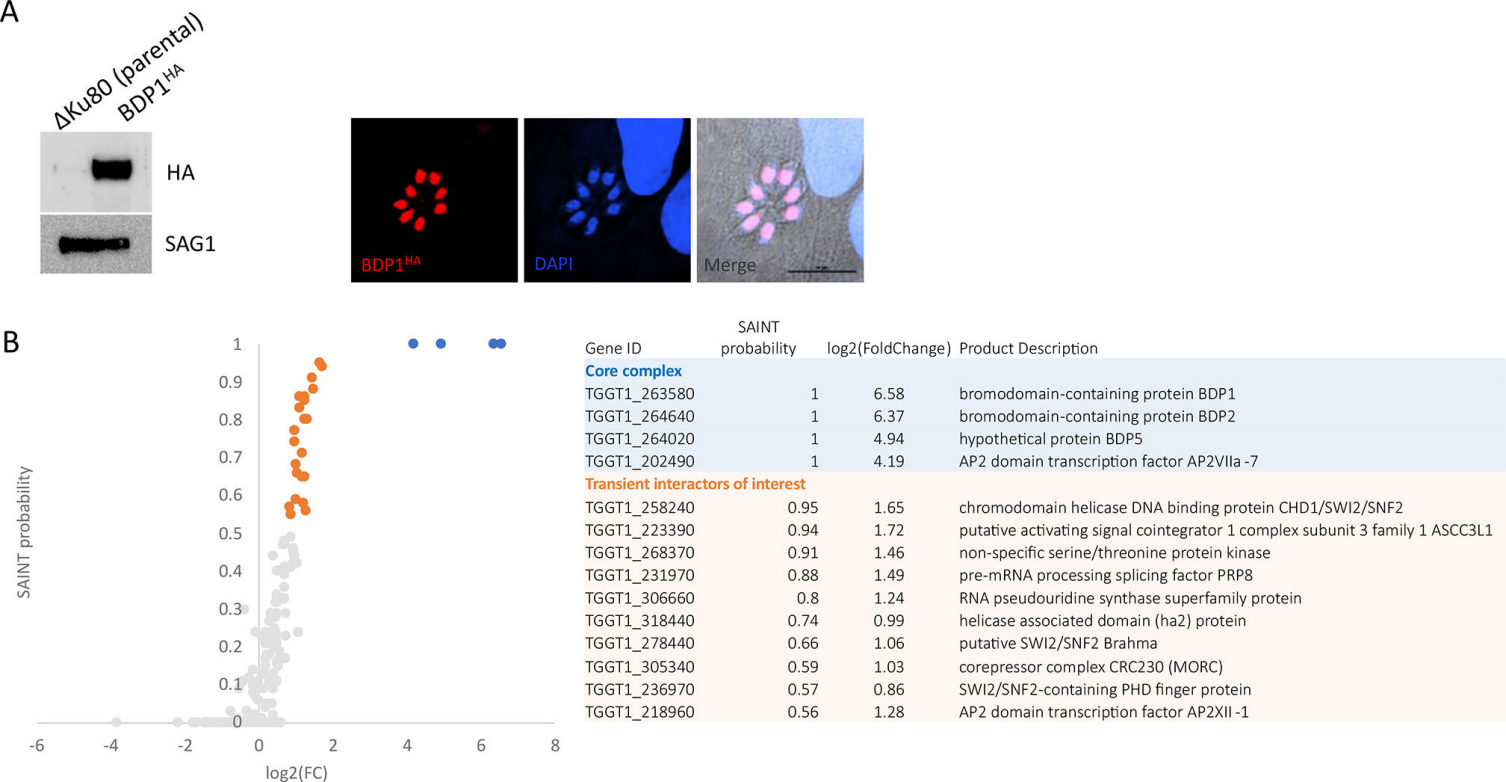
TgBDP1 is a nuclear protein that interacts with transcriptional and chromatin regulatory proteins. (**A**) TgBDP1 was tagged at the C-terminus with 3xHA. Western blotting of parasite lysate showed the tagged protein at the predicted size (81 kDa) with no signal detected in the parental line (Δku80). SAG1 was used as a loading control. Nuclear localization of tagged TgBDP1 was confirmed by IFA. (**B**) CoIPs of both TgBDP1^HA^ and parental lines were conducted, and enriched proteins identified by mass spectrometry. Significance Analysis of INTeractome (SAINT) probability scores are plotted against log2(fold change) from all three replicates. TgBDP1 and its most significant interactors are plotted in blue. Other highly probable interactors are plotted in orange. Scores and gene IDs are detailed in the adjacent table.

Bromodomain-containing proteins generally function as a component of a larger epigenetic regulatory complex. We sought to determine the function of TgBDP1 during tachyzoite proliferation by identifying TgBDP1-interacting proteins. A transgenic parasite line was generated in which the C-terminus of TgBDP1 was tagged with a 3xHA epitope tag. Western blotting and IFA confirmed the correct protein size (81 kDa) and nuclear localization of TgBDP1 (Fig. 4A). This parasite line was used for coIPs followed by mass spectrometry of the pulldown to identify TgBDP1-associated proteins.

**Fig 5:**
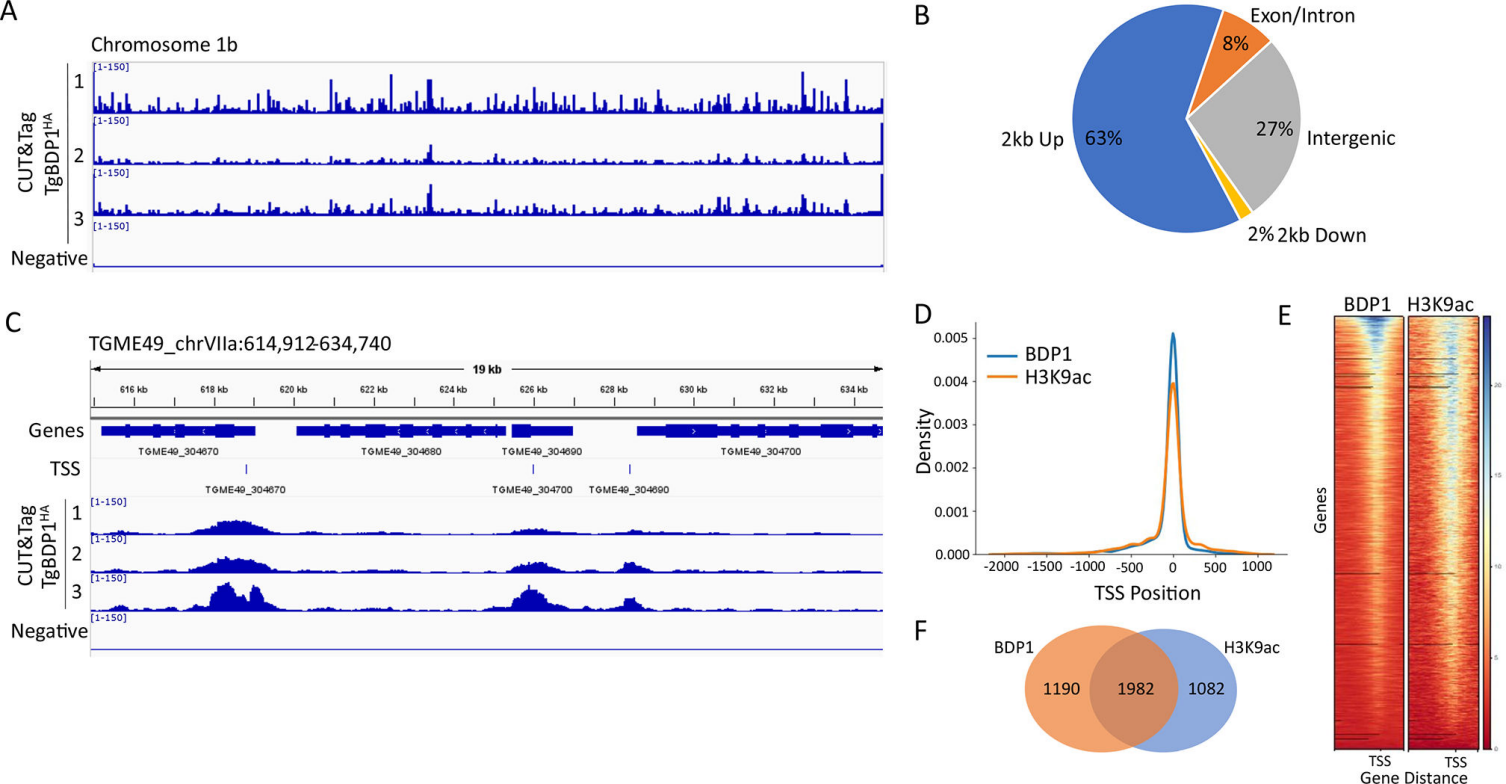
TgBDP1 binds upstream of many protein-coding genes. (**A**) Alignment of peak intensities of three replicates of TgBDP1 CUT&Tag and a negative control mapped to the *Toxoplasma* chromosome 1. The data range is the same between all four tracks. (**B**) A breakdown of the location of all TgBDP1 peaks relative to protein-coding genes. (**C**) Representative snapshot of TgBDP1 peaks aligning with transcriptional start sites of three genes on chromosome VIIa. (**D**) Density graph of TgBDP1 and H3K9ac peaks located –2 kb and +1 kb from TSS. (**E**) Heatmap of TgBDP1 and H3K9ac peak densities at gene distances from TSS. (**F**) Venn diagram of the number of genes with TgBDP1 and H3K9ac peaks at TSS.

Peptide counts from three independent replicates were submitted to REPRINT (https://reprint-apms.org/) (28) for SAINT analysis to identify the most highly significant proteins that interact with TgBDP1 (Fig. 4B). SAINT analysis evaluates total peptide counts between control and test samples and assigns a score (between 0 and 1) to each protein hit that describes the probability that a protein is a genuine interactor of TgBDP1. The most significant and abundant protein isolated alongside TgBDP1 with a SAINT score of 1 was the bromodomain protein TgBDP2 (Fig. 5B). Two other proteins were also assigned SAINT scores of 1, including the bromodomain-containing protein, TgBDP5, and the AP2 factor TgAP2VIIa-7 that contains a PHD and a SET domain. In addition to this core network of factors, 10 additional proteins with predicted nuclear localization were identified as potentially significant interactors (Fig. 4B; Table S1), including predicted transcription factors, chromatin remodeling factors, and RNA-associated proteins and enzymes involved in DNA, RNA, and chromatin-related pathways (Fig. 4B).

The significant enrichment but low abundance and diverse function of these TgBDP1-bound proteins suggests that TgBDP1 may be a component of multiple complexes involved in a variety of chromatin-related processes. These findings imply that TgBDP1 interacts with TgBDP2, together with TgBDP5 and TgAP2VIIa-7 to interact with many complexes with different biological functions.

### TgBDP1 is found at transcriptional start sites of active genes

We next addressed the occupancy of TgBDP1 across the *Toxoplasma* genome by using CUT&Tag. This technique has several advantages over traditional ChIP-seq and has provided high-quality results with human, mouse, and zebrafish cells (29). We adapted the technique for use with *Toxoplasma* parasites and used our transgenic TgBDP1^HA^ line to determine TgBDP1 genome-wide localization. Briefly, an anti-HA antibody was used to target transposases harboring sequencing tags to chromatin-bound TgBDP1. The transposase then cleaved DNA on either side of the TgBDP1 binding site, adding sequencing tags to the DNA ends. Tagged DNA fragments were amplified, sequenced, and mapped to the *Toxoplasma* genome. Our results were consistent between three independent replicates with little to no signal in the parental negative controls (Fig. 5A). To validate the CUT&Tag approach in *Toxoplasma,* we also performed the experiment with an antibody to acetylated lysine 9 on histone H3 (H3K9ac), a well-known active gene marker that is often found at TSS. The distribution of H3K9ac observed by CUT&Tag was consistent with previous ChIP-chip studies (30). An average of approximately 5,400 peaks for TgBDP1 binding sites was identified, the majority of which (63%) were located upstream of protein-coding genes (Fig. 5B and C; Table S2). Only 39% of TgBDP1 peaks located at transcriptional start sites coincided with H3K9ac peaks (Fig. 5D). These results suggest that TgBDP1 may bind or coincide with H3K9ac but not exclusively; it likely has roles independent of this particular acetyl mark. Our observation that TgBDP1 was bound upstream of open reading frames prompted us to compare TgBDP1 binding sites with predicted transcriptional start sites obtained from a recent study by Markus et al. (31). Of the 2,155 TgBDP1 peaks found within 2 kb upstream of protein-coding genes, a large proportion (42%) align directly with transcriptional start sites (Fig. 5C and D; Table S3). We also observed strong enrichment of TgBDP1 at the ends of chromosomes corresponding with regions of the telomeric repeats “TTTAGGG,” but TgBDP1 binding did not extend into subtelomeric regions as defined by Contreras et al. (32).

**Fig 6:**
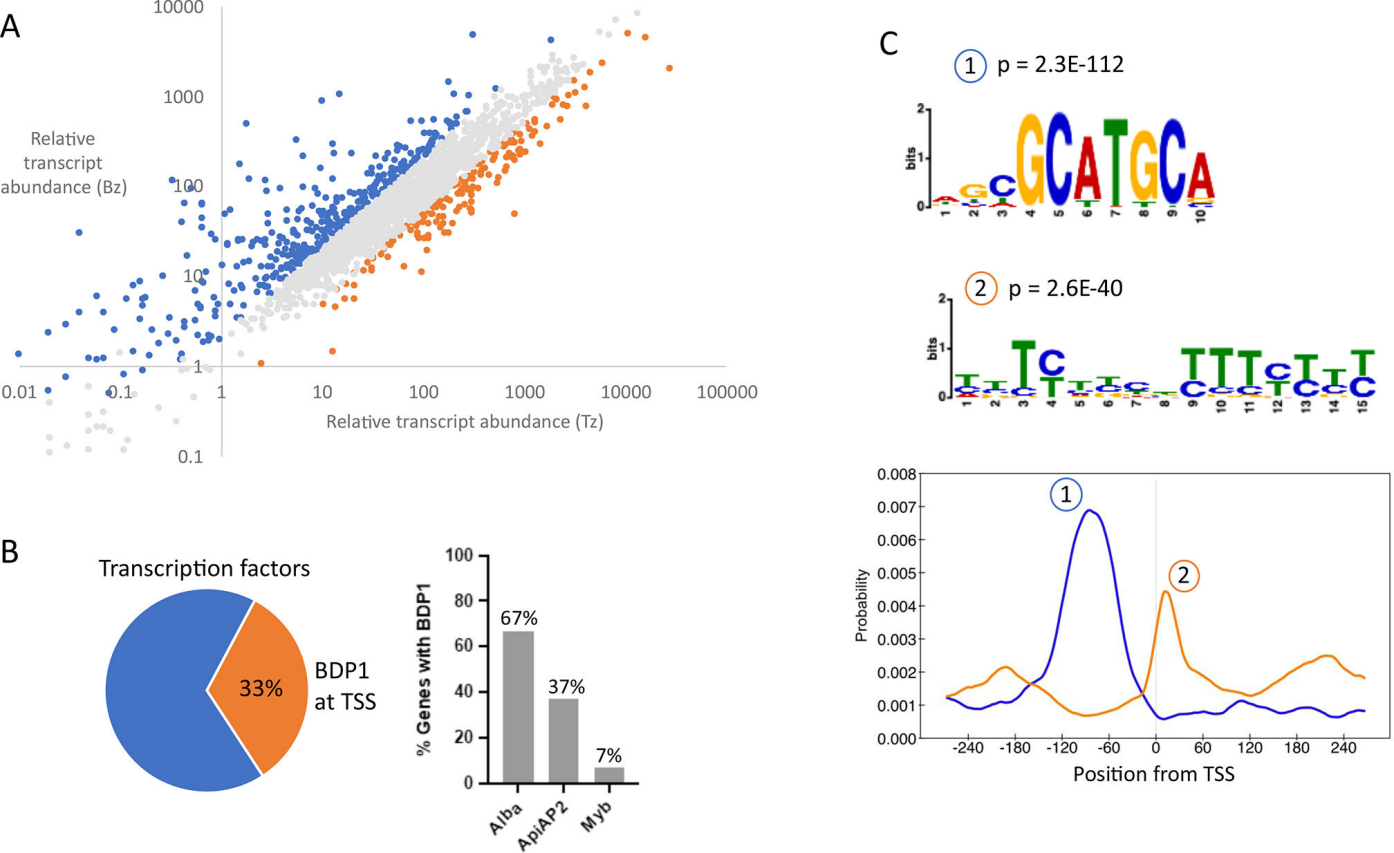
TgBDP1 is predominantly recruited to promoters of active genes. (**A**) Relative transcript abundance of putative TgBDP1 target genes in tachyzoites (*x*-axis) and after 48 h of bradyzoite induction (*y*-axis) (33). Blue markers, transcripts upregulated twofold or more; orange markers, transcripts downregulated twofold or more; gray: transcripts not significantly changed during bradyzoite differentiation. (**B**) Left: pie chart of the percentage of predicted transcription factor genes with (orange) and without (blue) TgBDP1 bound. Right: bar graph showing the percentage of each family of transcription factor genes bound by TgBDP1. (**C**) Motif analysis of TSS sequences associated with TgBDP1. Top: the two most significant motifs identified and their *P*-values. Bottom: location of each motif from TSS.

The expression profiles of TgBDP1 target genes were compared between tachyzoites and parasites undergoing bradyzoite differentiation. Using previously published transcriptomic data from Waldman et al. (33), we plotted the relative abundance of transcripts of TgBDP1-target genes during tachyzoite replication (*x*-axis) and early bradyzoite differentiation (*y*-axis) (Fig. 6A). Most TgBDP1-target genes are consistently expressed under both growth conditions (gray markers) suggesting that TgBDP1 is recruited to constitutively active genes. We also observed TgBDP1 binding to promoters of genes that are induced during bradyzoite formation (blue markers), suggesting that TgBDP1 may play a role in regulating chromatin structure to “poise” a gene for increased expression in response to environmental signals. However, a subset of TgBDP1-target genes are expressed at a low or undetectable level in both tachzyoites and early bradyzoites (lower left side of the plot), demonstrating that TgBDP1 binding within a gene promoter does not directly correlate with active or poised transcription, and indeed, TgBDP1 may act as a repressor on these genes.

Gene ontology (GO) enrichment analysis was performed on TgBDP1 target genes to determine if it is a regulator of specific functional pathways. TgBDP1 was bound to the promoter of 24% of all annotated *Toxoplasma* genes; so accordingly, functional enrichment analysis identified a diverse range of different biological processes that may be subject to TgBDP1 regulation. Those pathways that were significantly enriched include transcription and mRNA splicing, ribosomal formation and protein maturation, and metabolic processes associated with the mitochondrion. Manual inspection of the list of TgBDP1 target genes also revealed many transcriptional regulators that may be subject to TgBDP1 regulation. Of the 85 predicted transcription factors in the *Toxoplasma* genome, 33% were found to have TgBDP1 at their TSS, including 25 ApiAP2s and the myb regulator of bradyzoite formation, *tgbfd1* (Fig. 6B) (33). Supporting our observation that *tgbdp1*-knockdown parasites were defective in host cell invasion, 105 genes encoding microneme, rhoptry, and dense granule proteins are putative TgBDP1 targets.

TgBDP1 is recruited to many but not all active genes in the tachyzoite, so we performed motif enrichment analysis of TgBDP1-target gene promoters to identify any specific DNA sequence motifs that may contribute to TgBDP1 recruitment. MEME-ChIP analysis of sequence 250 bp upstream and downstream of the predicted TSS identified two DNA sequence features that were significantly enriched in target promoters (Fig. 6C). The motif GCATGCA (motif 1) and a degenerate pyrimidine-rich sequence (motif 2), which is enriched downstream of the TSS of selected genes, both of these motifs have previously been reported as characteristics of *Toxoplasma* TSSs (31, 34 - 36).

### Loss of TgBDP1 impacts parasite transcription

**Fig 7:**
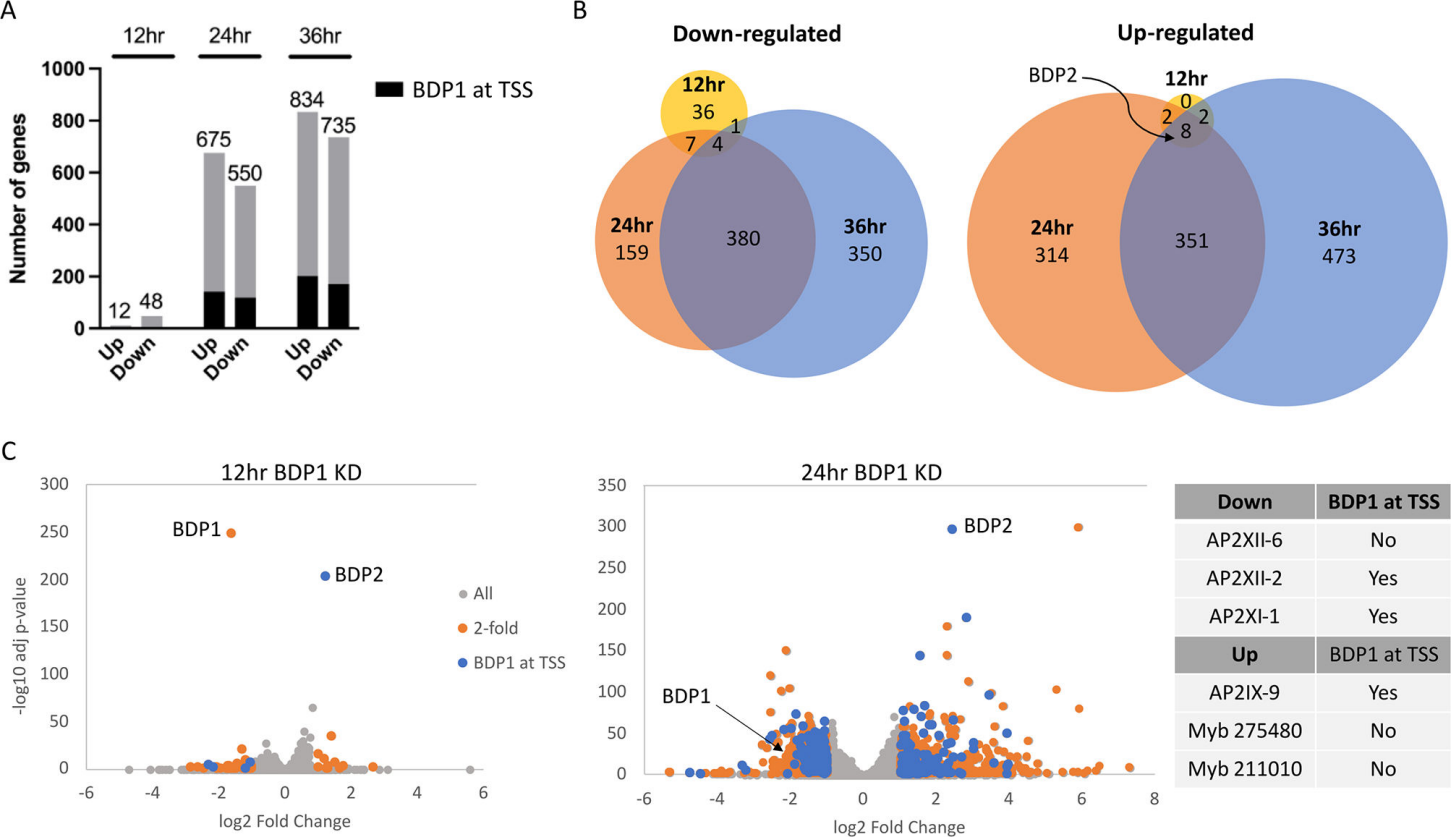
TgBDP1 downregulation causes global dysregulation of gene expression. (**A**) Bar graph depicting the number of genes up- and downregulated identified by RNA-seq in ^tet-myc^TgBDP1 parasites incubated for 12, 24, and 36 h with ATc. Black shading indicates the number of those genes with TgBDP1 found at the gene TSS from CUT&Tag analysis of TgBDP1 binding sites. (**B**) Venn diagrams of genes up- and downregulated between all three timepoints. TgBDP2 is the only gene differentially expressed at all three timepoints that is also bound by TgBDP1 at its TSS. (**C**) Volcano plots of differentially expressed genes in ^tet-myc^TgBDP1 knockdown parasites at 12 and 24 h. Genes up- or downregulated twofold or more are in orange and those also identified as TgBDP1 bound by CUT&Tag are in blue. The table shows specific transcription factors differentially expressed at 24 h post-knockdown.

Loss of TgBDP1 caused a defect in host cell invasion and an arrest in tachyzoite replication. As TgBDP1 appears to be a component of at least one epigenetic complex and is recruited to approximately 25% of predicted open reading frames, we investigated if the observed growth arrest during knockdown was due to global dysregulation of parasite transcription. Western blotting analysis of TgBDP1 during knockdown (Fig. 2D) indicates that TgBDP1 protein levels fall below 50% by 24 h and are almost undetectable by 36 h. To determine the essential contribution of TgBDP1 to transcriptional regulation, we performed RNA-seq at 12, 24, and 36 h after the addition of ATc. We observed a time-dependent effect of *tgbdp1* knockdown on the parasite transcriptome with a small number of genes impacted at 12 h but larger impacts observed at later timepoints (Fig. 7A). More than half of differentially expressed genes identified at 24 h were also dysregulated at 36 h (Fig. 7B). This observation, in addition to phenotypic data showing complete growth arrest at 36 h (Fig. 3), suggests that differentially expressed genes at 12 and 24 h likely represent more direct effects of *tgbdp1* knockdown, but by 36 h there are more indirect effects on parasite gene expression. Therefore, we focused our analyses on differentially expressed genes at the 12- and 24-h timepoints.

A small number of genes were significantly dysregulated by twofold or more at 12 h. Most significant was the upregulation of *tgbdp2*, the gene encoding TgBDP2, the binding partner of TgBDP1 (Fig. 7C; Table S4). Of the 48 significantly downregulated genes, TgBDP1 is recruited to the TSS of only four—ROP36 (TGME49_207610), an aspartyl protease expressed in sporulated oocysts (TGME49_272510) (37), a *T. gondii* family D protein (TGME49_313000) and a hypothetical protein (TGME49_243700). Apart from *tgbdp1* itself, only one of the genes that are significantly downregulated has a phenotype score <–1 (TGME49_211000); therefore, the downregulation of these genes is unlikely to contribute directly to parasite growth arrest. At this early timepoint in *tgbdp1* downregulation, there is very little impact on gene expression, aside from the significant upregulation of *tgbdp2,* which is likely a compensatory response to loss of TgBDP1.

A more dramatic impact on parasite transcription was observed by 24 h post *tgbdp1* knockdown. A total of 550 genes were downregulated twofold or more, only 118 of which are bound by TgBDP1 at their TSS (Fig. 7C; Table S3). GO enrichment analysis was performed on the lists of significantly dysregulated genes but did not identify any specific functional pathways that were enriched. Unexpectedly, a larger number of genes were upregulated in response to *tgbdp1* knockdown, the majority of which should peak in transcription levels during the chronic stage or sexual development in the cat intestine (Fig. S3). Of the 675 upregulated genes, 140 are also bound by TgBDP1 at the TSS. GO enrichment analysis of upregulated genes identified over fourfold (*P* = 0.01) enrichment of genes encoding surface proteins and invasion-related proteins from the SAG, microneme, dense granule, and rhoptry families. Although 105 invasion genes were identified by CUT&Tag to be associated with TgBDP1, only 19 of those were upregulated. Therefore, the majority of dysregulated invasion genes are likely indirectly regulated by TgBDP1. These results suggest that loss of TgBDP1 influences the expression of other critical transcriptional activators or repressors; TgBDP1 is bound to the TSS of many putative transcription factors (Fig. 6B), and several transcription factors are dysregulated during *tgbdp1* knockdown (Fig. 7C). It is important to note that the knockdown cultures from which RNA was extracted for analysis were inoculated from seed cultures that had been cultured for 36 h and were beginning to egress. Since egressing parasites are stalled in the G1 stage of the cell cycle, the parasites may have been partially synchronized, and differences in transcript levels between the control and the knockdown timepoints may be overlaid with cell cycle effects.

The most consistent effect of *tgbdp1* knockdown is the sustained upregulation of *tgbdp2*. This is the only gene identified at all three timepoints that was differentially expressed that also had TgBDP1 bound at the TSS, per CUT&Tag analysis (Fig. 7B). It remains unclear whether residual TgBDP1 is directly involved in upregulating *tgbdp2* expression or if loss of TgBDP1 triggers an indirect compensatory response. Overall, the large number and functional diversity of both up- and downregulated genes impacted during *tgbdp1* knockdown supports a global chromatin regulation function for TgBDP1 rather than merely a transcriptional activator. Furthermore, the subset of transcription factors that are directly and indirectly impacted by TgBDP1 points to this bromodomain and its complex(es) as key players of *Toxoplasma* gene expression.

## DISCUSSION

Histone acetylation is associated with activation of gene expression. Specific histone acetylation marks typical for gene activation, such as H3K9Ac and H4K8Ac, K12Ac and K16Ac are enriched at active gene promoters in *Toxoplasma* tachyzoites (8, 10, 30). In addition to altering chromatin structure to facilitate transcription, histone acetylation serves to recruit regulatory complexes to specific loci through the action of reader modules, such as bromodomains, although the precise function of most bromodomain-containing proteins in *Toxoplasma* gene regulation has yet to be determined. To understand the contribution of bromodomain proteins in *Toxoplasma* in mediating signals between histone acetylation marks and transcription initiation complexes, we investigated the role of a protein unique to alveolates and conserved within the Apicomplexa, TgBDP1. Based on reports on the *P. falciparum* BDP1 homologue (16, 17), we initially hypothesized that TgBDP1 was a transcriptional activator that binds to activating acetylation marks on histone tails to recruit AP2 proteins and other transcription factors to active gene promoters. This was supported by both our CUT&Tag and proteomic analysis that found TgBDP1 bound to chromatin at the predicted TSS of many active genes in tachyzoites and associated with putative transcription factors and epigenetic regulators. However, analysis of global transcription revealed that more genes were upregulated than downregulated during ablation of TgBDP1, indicating a more complex role for this regulatory factor. Furthermore, we did not find a correlation between dysregulated genes and those that have TgBDP1 bound at the TSS, suggesting that much of the impact on gene expression that we observed is due to indirect effects of *tgbdp1* knockdown. TgBDP1 may influence transcription through regulation of transcriptional activators and repressors or via other, non-chromatin binding events, such as associating with acetylated transcription machinery.

Proteomic analysis of the TgBDP1 interactome supports multiple regulatory functions for the core TgBDP1/2/5 complex. We identified a consistent association between the three bromodomain-containing proteins and the AP2 factor AP2VIIa-7, which contains a methyltransferase domain in addition to the AP2 domain. Our proteomic approach also detected transient interactions between TgBDP1 and three sWI/SNF nucleosome remodelers, implying a role for this complex in the regulation of chromatin structure. Modulation of local chromatin structure can serve to both facilitate or repress transcription. TgBDP1 acting as a major regulator of chromatin structure would explain its apparent contrary influence on gene expression. We should also consider the presence of two isoforms of TgBDP1 (Fig. S1) that may define different complex compositions and functionalities. Our analysis also detected an interaction between TgBDP1 and TgMORC, one of the major regulators of parasite stage-specific gene expression. TgMORC is required for recruitment of the HDAC3 repressor complex to maintain silencing of specific genes during the tachyzoite stage (38). The association of TgBDP1 with TgMORC hints at a role for TgBDP1 in gene silencing and may explain the large number of upregulated genes during *tgbdp1* knockdown (almost half of the genes (307 out of 676) upregulated during *tgbdp1* knockdown are TgMORC target genes), but further study of this interaction is necessary to understand the functional relevance to parasite transcriptional regulation. TgMORC transcript levels were also slightly increased during *tgbdp1* knockdown (1.56-fold) but were excluded by our cutoffs. If this increase in TgMORC transcript levels results in functionally relevant increase in TgMORC protein, then this may additionally explain the downregulation of genes that do not have TgBDP1 bound at their TSS. Another interactor with the TgBDP1 core complex was TGGT1_235420, a protein of unknown function that localizes to the nucleus. Although the function of this protein is unknown, it is essential for tachyzoite survival in both *Toxoplasma* and the related coccidian *Neospora caninum* (39).

Many of the genes that are upregulated in response to *tgbdp1* knockdown are more highly expressed in bradyzoites or sexual stages compared with tachyzoites (Fig. S3). It is unclear if TgBDP1 is directly repressing the expression of these genes or if it indirectly represses stage-specific genes by regulating transcriptional factors required for maintenance of appropriate stage expression. Transcription factors dysregulated during *tgbdp1* knockdown include three AP2 factors that are significantly downregulated (AP2XI-1, AP2XII-2, and AP2XII-6). One of these, AP2XII-2 is a cell cycle-regulated protein associated with the TgMORC/HDAC3 repressor complex (38). Sustained AP2XII-2 expression may be required during tachyzoite growth to target the MORC complex to sexual or bradyzoite stage genes and maintain tachyzoite proliferation. Indeed, Srivastava and colleagues demonstrated that the knockdown of AP2XII-2 results in slowed tachyzoite replication and increased cyst formation (39, 40). We also observed significant upregulation of genes encoding three putative DNA binding proteins: two myb domain-containing proteins and the AP2 factor AP2IX-9. The functions of the two myb domain proteins are unknown; however, they are both consistently expressed across all life cycle stages, suggesting that they may have housekeeping functions, and their upregulation may be a compensatory response to loss of TgBDP1. AP2IX-9 is a repressor of bradyzoite commitment that is normally upregulated in response to bradyzoite induction conditions (41). TgBDP1 is bound to the TSS of AP2IX-9 in unstressed tachyzoites, so it is unclear how loss of TgBDP1 contributes to upregulation of AP2IX-9. There are a couple of possibilities for TgBDP1’s influence on *ap2ix-9* expression; TgBDP1 maintains repression of *ap2ix-*9 directly through its interactions with TgMORC and that loss of TgBDP1 derepresses *ap2ix-9*. Alternatively, TgBDP1 poises *ap2ix-9* for expression, and upregulation of *ap2ix-9* is a direct response to the stress induced in the parasites by loss of TgBDP1. Another major regulator of parasite stage transition TgBFD1, which promotes upregulation of bradyzoite genes during the initial stages of differentiation into tissue cysts was also slightly upregulated (1.83-fold). However, since TgBFD1 appears to be primarily regulated at the translational level, and the protein that drives translation of TgBFD1, TgBFD2 (42) is not significantly increased during *tgbdp1* knockdown, it is unclear if this slight increase in *tgbfd1* transcript leads to increased protein levels.

One family of proteins upregulated during *tgbdp1* knockdown is the SAG1-related (SRS) family of surface proteins. This mirrors a recent report that evaluated gene expression patterns during knockdown of TgBDP5, a bromodomain protein that is also a component of the TgBDP1 core complex (43). Many of those genes upregulated during *tgbdp1* knockdown peak in expression level during sexual stages or the bradyzoite tissue cyst (44). A single-cell RNA-seq analysis revealed that during normal tachyzoite growth, expression of many “stage-specific” SRS surface antigens is variable between individuals within a population of clonally derived parasites (45). Although the function of these surface proteins is unknown, the authors of these studies speculate that they may contribute to antigenic variation. In *P. falciparum*, many of the variable surface antigens *var, stevor,* and *rifin* were derepressed during knockdown of either the BDP1 (PfBDP1) or BDP5 (PfBDP7) homologues, and there is strong evidence for a role of this protein complex in maintaining mutually exclusive expression of a single surface antigen by repressing other surface antigen genes (18). Although we did not observe TgBDP1 bound at all *srs* gene loci that were upregulated during *tgbdp1* knockdown, it remains an intriguing possibility that the TgBDP1/2/5 complex contributes to a form of antigenic variation or virulence factor regulation in *Toxoplasma* by mediating stage-specific repression of these surface proteins.

TgBDP1 is recruited to over a thousand specific sites in the parasite genome. A large proportion of binding sites correlate with both predicted TSSs (31) and histone marks linked with transcriptional initiation, but it is unclear how the TgBDP1 complex is recruited to these target binding sites. Interactome analysis identified a total of three bromodomain-containing proteins and a putative DNA binding protein (AP2VIIa-7) in the core complex indicating that this recruitment is mediated by recognition of a DNA sequence motif and/or histone modifications. Our analysis of the sequences around TgBDP1 TSS binding sites identified two significantly enriched sequence features, one of which, the GCATGC motif, has been reported previously in *Toxoplasma* (31, 34, 35) as an enriched motif at TSSs in tachyzoites, so is unlikely to be a specific motif to recruit the TgBDP1 complex and rather a general feature of *Toxoplasma* gene promoters. It is likely that interactions between the three bromodomains in the complex and histone acetyl marks are more important mediators of complex recruitment to the chromatin. Determining the histone marks that are preferentially bound by the TgBDP1 bromodomain and the two interacting bromodomain-containing proteins TgBDP2 and TgBDP5 will be an important next step in understanding the function of this complex. We observed a strong correlation between TgBDP1 target sites and the histone H3K9Ac modification, suggesting that this may be one of the histone marks that is “read” by one or more of the bromodomains in the TgBDP1/2/5 complex. However, TgBDP1 was not located at every H3K9Ac-enriched site, indicating that another histone mark dictates TgBDP1/2/5 complex recruitment to the chromatin. The *P. falciparum* homologue, PfBDP1, did not display a strong affinity for acetylated histone H3 peptide, even when multiple lysine residues were acetylated, suggesting that acetylated H3 is not important for complex recruitment. However, PfBDP1 has high binding affinity for acetylated histones H4, H2b.Z, and H2a.z (24, 46), particularly when multiple acetyl marks are present, suggesting that the PfBDP1/BDP2 complex is recruited to highly acetylated chromatin rather than a specific acetylated histone residue. Additional studies will be needed to determine if this is also the case in *Toxoplasma*. Furthermore, it is probable that the ankyrin repeats of TgBDP1 mediate another important TgBDP1–protein interaction. The ankyrin repeats of the human methyltransferases G9a and G9a-like are reader modules of mono- and di-methylated H3K9 (47), so the ankyrin repeats of TgBDP1 may function in a similar manner to target the complex to modifications on chromatin. We also cannot discount the possibility that the bromodomain proteins may recognize a non-histone acetylated protein, of which many have been reported in *Toxoplasma* in previous studies (48 - 50). The regulatory implications of acetylation marks on non-histone proteins in the nucleus have not been investigated, but it is likely that this process contributes to another level of transcriptional regulation.

TgBDP1 also appears to be recruited to telomeric regions. Given that *Toxoplasma* telomeres are heterochromatic (32, 51) and, therefore, the histones are hypoacetylated, it is unclear how TgBDP1 might be recruited to these regions. The extent of TgBDP1 enrichment at the telomeres may be artifactual due to the highly repetitive sequence structure of the telomeres that challenges accurate mapping of sequence reads, so further studies will be needed to confirm TgBDP1 binding to these regions.

Several aspects of TgBDP1 biological function parallel to that of the *P. falciparum* homologue PfBDP1, including interacting closely with additional bromodomain-containing proteins, binding at TSSs, and regulating gene expression (16, 18). However, we identified key differences that suggest distinct roles between species. Knockdown of PfBDP1 negatively impacted parasite invasion but with no effect on replication or overall fitness, whereas TgBDP1 knockdown resulted in major defects to replication and ultimately parasite death. We found that TgBDP1 associates with a larger cohort of epigenetic regulators compared with the PfBDP1-associated proteins and regulates a larger proportion and more functionally diverse sets of genes, suggesting TgBDP1 has additional functions beyond those reported for the *P. falciparum* homologue.

We show that while TgBDP1 has a highly conserved bromodomain, it is otherwise divergent from human, yeast, and plant bromodomain proteins. Only apicomplexans and a small number of other alveolates possess a homologous protein. As this essential protein is highly conserved among the human pathogens within the phylum Apicomplexa, it serves as a promising candidate for drug development that warrants more in-depth study.

## MATERIALS AND METHODS

### Cell culture

*T. gondii* tachyzoites of RHΔHXΔKu80 and TATiΔKu80 backgrounds (52, 53) were maintained in human foreskin fibroblast (HFF) cells and Dulbecco’s Modified Eagle Medium supplemented with 1% fetal bovine serum. Cells were cultured in a humidified incubator at 37°C with 5% CO2. ^tet-myc^TgBDP1 and TgBDP1-HA cell lines were maintained in media containing 1 uM pyrimethamine.

### TgBDP1 sequence analyses

The predicted TGME49_283580 (TgBDP1) genomic DNA, mRNA, and protein sequences were obtained from ToxoDB (https://toxodb.org) (25). BLASTp analyses using the TgBDP1-predicted protein sequence were conducted in both the NCBI and VEuPathDB databases to identify homologues. Protein sequences from BDP1 homologues were aligned and evolutionary analysis performed using the maximum likelihood method in MEGA11 (54). Clustal Omega (EMBL-EBI) was used to align bromodomain sequences. The structure of TgBDP1’s bromodomain was predicted using I-TASSER and overlayed with the experimentally determined structures of the human B2AZB bromodomain (PDB 5DYU) and the *P. falciparum* PfBDP1 bromodomain (PDB 7M97) using Chimera (https://www.rbvi.ucsf.edu/chimerax) (55). The ToxoDB genome browser, JBrowse, was used to visualize predicted introns and nanopore mRNA sequencing reads from the Lee et al. data set (26). To confirm TgBDP1 mRNA transcript sequences, RNA was harvested from RHΔHXΔKu80 parasites and cDNA synthesized using the Omniscript RT kit (Qiagen 205113) with *tgbdp1* specific primers 5′TTCAAAGATATGTCCACCCTCG and 5′CCTTACATCAGCAGACCTGC. The resulting cDNA was used to amplify tgbdp1 transcripts with primers 5′AGTGAATTCGAGCTCGGTACCATGTCGACTGGCGCGAGTG and 5′TGCATGCCTGCAGGTCGACTCTAGATTAAGCTCCACGTGATTCTCCG, which were then cloned into a pUC19 vector. Plasmid DNA was isolated from six different bacterial clones and sequenced.

### Generation of *TgBDP1* knockdown (^tet-myc^TgBDP1)

Inducible knockdown of the *tgbdp1* gene was accomplished by replacing the endogenous *tgbdp1* promoter with a tetracycline regulatable *tgsag4* promoter and adding a 3xmyc tag to the 5′end of the *tgbdp1* gene. A 2,100-bp region of genomic DNA directly downstream of the *tgbdp1* start codon was amplified with primers 5′catctccgaggaggacctgagatctTCGACTGGCGCGAGTGTG and 5′TACGATGCGGCCGCcgatacatctgggcttgcc from TATiΔKu80 genomic DNA. The DHFR-tet07Sag4-3xMyc-CEP250 plasmid (kindly provided by MJ Gubbels) was digested with BglII and NotI, and the HiFi DNA Assembly kit (NEB E5520S) was used to insert the *tgbdp1* fragment. The final DHFR-tet07Sag4-3xMyc-tgbdp1 plasmid was verified by sequencing. One hundred micrograms of plasmid was linearized with NotI and transfected into TATiΔKu80 (TATi) parasites (kindly provided by MJ Gubbels) by electroporation in cytomix (56). Parasites were cultured with 1 uM pyrimethamine for selection and cloned by limiting dilution. PCR of genomic DNA with primers P1 5′GCTAATCTCCGAGGAAGACTTG and P2 5′TGGCCTGCTCTCGTTTCAC was used to confirm correct integration. PCR with primers P2 5′CGATTGCCTCTCCCTCAAGTCC and P3 5′TCTCGACCTCTTCGCGTACG confirmed disruption of the endogenous promoter.

### Generation of endogenously tagged TgBDP1 (TgBDP1^3xHA^)

A 3xHA epitope tag was introduced at the 3′ end of the endogenous *tgbdp1* gene. A 2,131-bp region of genomic DNA upstream of the *tgbdp1* stop codon was amplified with primers 5′tacttccaatccaatttaattaaTGAGCAAGTGAGGCAAGC and 5′cctccacttccaattttaattaaAGCTCCACGTGATTCTCC and inserted using the HiFi DNA assembly kit into pLIC-3xHA-DHFR cut with PacI. The final pLIC-TgBDP1-3xHA-DHFR plasmid was verified by sequencing. An amount of 100 μg of plasmid was linearized with AflII and transfected into RHΔHXΔKu80 (ΔKu80) parasites by electroporation in cytomix. Parasites were cultured with 1 uM pyrimethamine for selection and cloned by limiting dilution.

### Immunofluorescence assays

Parasites were inoculated into 24-well plates of confluent HFFs containing coverslips and cultured approximately for 24 h (±ATc), then fixed with 4% paraformaldehyde, and permeabilized with Triton X-100 in 3% bovine serum albumin (BSA). Samples were blocked in 3% BSA, and primary and secondary antibodies were diluted in 3% BSA. Primary antibodies anti-myc (Invitrogen 132500) and anti-HA (Roche 27573500) were diluted 1:2,000, and secondary antibodies anti-mouse Alexa Fluor 594 (Thermo Fisher Scientific A11005) and anti-rat Alexa Fluor 594 (Thermo Fisher Scientific A11007) were diluted 1:5,000. After antibody incubations, samples were incubated with DAPI (Invitrogen D1306) in 3% BSA and coverslips mounted to slides with Vectashield mounting medium (Vector Laboratories H1000). Slides were visualized and imaged with a Nikon A1R laser scanning Confocal Fluorescence Microscope and NIS-Elements software.

### Western blotting

Protein was isolated from parasites by resuspending harvested parasites in RIPA lysis buffer supplemented with a protease inhibitor cocktail (Research Products International Corp P506001). Samples were then sonicated using a QSonica Q800R3 at 50% amplitude for 2 min. Insoluble material was pelleted and removed. Protein concentrations of lysates were determined using a BCA protein assay kit (Thermo Fisher Scientific 23227), and 50 ug of protein was used for Western blotting. Protein samples were separated by SDS-PAGE in 4%–15% Bis-Tris gels with MOPS buffer and transferred to nitrocellulose membrane. Membranes were blocked in 5% non-fat milk and incubated in primary and secondary antibodies diluted in 5% non-fat milk. The following antibodies were used: anti-myc-HRP (Santa Cruz sc-40) diluted 1:100, anti-HA (Roche 27573500) diluted 1:2,000, anti-rat-HRP (GE NA935) diluted 1:2,000, anti-p30 (SAG1) (Invitrogen MA183499) diluted 1:2,000, and anti-mouse-HRP (GE NA931) diluted 1:2,000. Pierce Enhanced Chemiluminescence (ECL) detection reagent (Thermo Fisher Scientific 32109) and a BioRadV3 Chemidoc Imager were used to visualize blots.

### Quantitative RT-PCR

Total RNA was harvested from ^tet-myc^TgBDP1 parasites 36 h post-inoculation and that had been cultured ±ATc 1 uM ATc for 12, 24, or 36 h. Parasites were pelleted and resuspended in 1 mL TRI Reagent (MilliporeSigma T9424). RNA was isolated by phenol:chloroform extraction and isopropanol precipitation followed by genomic DNA removal using the Turbo DNA-free kit (Invitrogen AM1907). Three micrograms of RNA were used to synthesize cDNA with the Omniscript RT kit using oligo dT primers (Qiagen 205113). The resulting cDNA was diluted 1:2 and used for real-time PCR with Power SYBR Green (Thermo Fisher Scientific 4367659) and Applied Biosystems 7500 real-time PCR system. *tgbdp1* was amplified with primers 5′CACATCCTCAGCAATTCCTTAAG and 5′GCGAGGACACTGTAGATCTTG, and *tgtuba1* (used for normalization) was amplified with primers 5′GATGCCCTCTGACAAGACC and 5′CATCCTCTTTCCCGCTGATC. The delta delta Ct method was used to quantify changes in gene expression compared with −ATc samples, and 2^−ddct^ was used to calculate fold change. Data from three independent replicates were statistically analyzed using one-way analysis of variance and Dunnett’s multiple comparisons test in GraphPad Prism Version 9.3.1 for MacOS, GraphPad Software, San Diego, CA, USA, www.graphpad.com.

### Toxoplasma growth assays

Plaque assays were done to assess the effect of TgBDP1 knockdown on *Toxoplasma* growth as previously described (56). Briefly, 200 parasites of the ^tet-myc^TgBDP1 and parental (TATi) parasite lines were inoculated into 12-well plates of confluent HFFs in media ±ATc and cultured for 6 d. Cells were then fixed in methanol, stained with crystal violet, and imaged with an Invitrogen EVOS M7000 microscope. The area of the plaques per well (area of host cell lysis) was quantified from the images using ImageJ software and percentage of host cell lysis compared with −ATc calculated. An unpaired *t*-test from three independent experiments was performed using GraphPad Prism.

*Toxoplasma* red/green invasion assays were performed as previously described (57). ^tet-myc^TgBDP1 and TATi parasites were cultured ±ATc for 36 h, at which point intracellular parasites were harvested and counted. Parasites and 12-well plates of HFFs containing coverslips were chilled on ice and 1 × 10^6^ parasites inoculated per well, remaining on ice for 15 min. The inoculated plate was then incubated in a 37°C water bath for 1 min before moving to the 37°C incubator. Plates were incubated for 2 h and then washed to remove extracellular parasites. Cells were fixed with 3% paraformaldehyde, blocked with 3% BSA, and incubated with 1:1,000 dilution of mouse anti-P30 (SAG1) primary antibody (Invitrogen MA183499). Cells were then permeabilized and incubated with 1:1,000 dilution of rabbit anti-Toxoplasma primary antibody (Invitrogen PA17252) followed by a final incubation with secondary antibodies goat anti-mouse Alexa Fluor 488 (1:5000) (Thermo Fisher Scientific A11001) and goat anti-rabbit Alexa Flour 594 (1:5,000) (Thermo Fisher Scientific A11012). A Zeiss Axioplan 2 fluorescent microscope was used to visualize over 1,000 parasites per treatment group. Red-only parasites were designated intracellular, while dual color parasites (red and green) were considered extracellular. The percentage of intracellular parasites was calculated, and an unpaired *t*-test between −ATc and +ATc groups for three independent experiments performed.

Doubling, or replication, assays were used to determine *Toxoplasma* replication rate. ^tet-myc^TgBDP1 and TATi parasites were cultured ±ATc for 24 h, at which point intracellular parasites were harvested and inoculated into a 12-well plate of confluent HFFs in media ±ATc. Two-hour post-inoculation media and extracellular parasites were removed, and fresh media ±ATc added. Wells were fixed with Hema3 fixative 12-, 24-, and 36-h post-inoculation and then stained with Hema3 Staining Solutions I and II. The number of parasites per vacuole was counted for 100 vacuoles. Three independent experiments were conducted.

### Co-immunoprecipitation

CoIPs and mass spectrometry were used to identify TgBDP1-interacting proteins in TgBDP1-HA parasites, with ΔKu80 parasites used as a negative control. For each sample, parasites were cultured for 36 h and 8 T-150s of intracellular parasites harvested. Nuclei were harvested by resuspending cells in 1 mL lysis buffer A [10 mM KCl, 10 mM HEPES pH 7.4, 0.1% NP-40, 10% glycerol, and cOmplete protease inhibitor cocktail (Roche 04693159001)], incubated on ice 5 min then pelleted at 10,000 × *g* for 10 min at 4°C. The nuclei pellet was resuspended in 500 uL lysis buffer B (400 mM KCl, 10 mM HEPES pH 7.4, 0.1% NP-40, 10% glycerol, and cOmplete protease inhibitor cocktail), vortexed for 30 min at 4°C, and centrifuged at 10,000 × *g* for 10 min at 4°C. For each 500 uL nuclear supernatant, 50 uL of prewashed anti-HA magnetic beads (Thermo Fisher Scientific 88837) was added, and samples rocked at 4°C overnight. Protein-bound anti-HA magnetic beads were washed five times in coIP buffer (0.025M Tris, 0.15M NaCl, 0.001M EDTA, 1% NP-40, 5% glycerol, and cOmplete protease inhibitor cocktail), then resuspended in 45 uL 2× LDS buffer and 8% beta-mercaptoethanol, and boiled 10 min. Samples were run on a 4%–12% Bis-Tis gel in MOPS buffer, and the gel stained with Coomassie G-250 for 1.5 h.

Protein samples were recovered by isolating four gel bands encompassing the entire lane that were then processed via in-gel digestion and analyzed by LC-MS and LC-MS-MS as described previously (58). Briefly, a 1-uL aliquot of the digestion mixtures was injected into a Dionex Ultimate 3000 RSLCnano UHPLC system with an autosampler (Dionex Corporation, Sunnyvale, CA, USA), where it was then separated in a 100 µm × 15 cm capillary packed with Dr. Maisch ReproSil-Pur C18-AQ, r13.aq (120 Å; 3 µm), at a flow rate of ∼450 nL/min. The eluant was connected directly to a nanoelectrospray ionization source of an LTQ Orbitrap XL mass spectrometer (ThermoFisher Scientific). LC-MS data were acquired in a data-dependent acquisition mode, cycling between an MS scan (m/z 315–2,000) acquired in the Orbitrap, followed by collision-induced dissociation analysis on the three most intensely multiply charged precursors acquired in the linear ion trap. The LC-MS/MS data were processed by PAVA bioinformatic program to generate the centroided peak lists of the CID spectra and searched against a database that consisted of the Swiss-Prot protein database (version 2021.06.18; 53/565,254 entries searched for *Toxoplasma Gondii*), using the Batch-Tag program module of the Protein Prospector bioinformatic package from the University of California, San Francisco, CA, USA (version 6.3.1). A precursor mass tolerance of 20 ppm and a fragment mass tolerance of 0.6 Da were used for protein database search (trypsin as enzyme; one missed cleavage; carbamidomethyl [C] as a constant modification; acetyl [protein N-term], acetyl + oxidation [protein N-term *M*], Gln ->pyro-Glu [N-term Q], Met-loss [protein N-term *M*], Met-loss +acetyl [protein N-term *M*], and oxidation [*M*] as variable modifications). Protein matches were reported with a Protein Prospector protein score ≥22, a protein discriminant score ≥0.0, and a peptide expectation value ≤0.01 (59). Data are available via ProteomeXchange with the identifier PXD038848. Potential TgBDP1-associated proteins were identified as those with peptide counts at least twofold higher in TgBDP1-HA samples compared with ΔKu80, present in at least two out of three independent experiments and with a predicted or verified nuclear localization. SAINT analysis was performed using REPRINT (https://reprint-apms.org/) with default settings (28).

### Cleavage under targets and tagmentation

CUT&Tag was used to identify the genomic localization of TgBDP1^3xHA^. Based on protocols and findings from the Henikoff lab (29), we modified the technique for use with *Toxoplasma* tachyzoites. For each sample, intracellular parasites cultured for 36 h were harvested from 1 T-75, syringe lysed, filtered through a 3 um filter and counted. Ten million (1 × 10^7^) parasites were centrifuged 2,000 × *g* for 10 min, and the parasite pellet used directly with the CUT&Tag-IT Assay Kit (Active Motif 53160). Our experimental sample used TgBDP1^3xHA^ parasites and 1 uL of a 1:50 dilution of the rabbit anti-HA primary antibody (Cell Signaling 3724T). The negative control sample used ΔKu80 parasites (the parental line) with the same primary antibody conditions. Samples were incubated with primary antibody overnight at 4°C. The remainder of the kit protocol was followed exactly, and unique indexed primers were used for each sample. Three biological replicates were done for both TgBDP1^3xHA^ and ΔKu80 parasites. Due to small cell number and low amount of input DNA needed for this technique, negative controls often result in very little to no DNA and are therefore not able to be sequenced. This was the case for one out of three negative control replicates. A single positive control sample was processed in parallel to confirm that our technique was successful. We used an anti-H3K9ac antibody (Active Motif 39917) with TgBDP1^3xHA^ parasites to identify the highly abundant H3K9ac mark throughout the *Toxoplasma* genome, which has previously been done using ChIP-chip (30).

Indexed libraries for each sample were analyzed by TapeStation, pooled and run on NextSeq 500/550 High Output (75 cycles) flow cell to generate paired end reads. Demultiplexing of the reads was performed with bcl2fastq version 2.20.0 and processed with cutadapt v3.4 (60) to filter sequencing adapters from the 3' end of reads, and any reads with fewer than 30 base pairs were removed. Remaining reads were mapped to version 52 of the Toxoplasma gondii ME49 reference downloaded from ToxoDB (25). The mapping was performed with HISAT2 v 2.2.1 (61) using the following parameters “--no-discordant--no-spliced-alignment --phred33 --no-unal --nomixed.” Unmapped reads were removed with samtools 1.9 (62). For each sample, peaks were then called using the callpeak command within MACS3 3.0.0a6 (63), with the parameters “-g 6.e7 -B -q 0.01.” A custom python script was used to associate predicted peaks with genes defined in version 52 of the ME49 reference and TSS identified in *Toxoplasma gondii* (31). A heatmap of mapped reads in relation to these TSSs was generated using the computeMatrix and plotHeatmap commands from deepTools version

3.5.0 (64). DNA motif analysis was performed using MEME-ChIP (65) with default settings and sequences 250 bp upstream and downstream of TSS with TgBDP1 associated peaks.

### RNA-sequencing (RNA-seq)

^tet-myc^TgBDP1 parasites were cultured 36 h and treated −ATc or +ATc for 12, 24, or 36 h. Intracellular parasites were harvested from two T-150s for each sample, syringe lysed, filtered through a 3-um filter and pelleted. The Qiagen RNeasy Plus Mini Kit was used to isolate RNA per the manufacturer’s instructions. Library preparation was completed with the KAPA mRNA HyperPrep Kit (Illumina® Platforms). Sequencing was completed at the Hubbard Center for Genome Studies on an Illumina NovaSeq 6,000 platform to produce 250 bp paired-end reads. Raw-sequencing data were demultiplexed using bcl2fastq v1.8.4 (Illumina). Read quality was examined with FASTQC v0.11.9. Adapters and low-quality sequences were trimmed from the reads using Trimmomatic V0.32 (66) with default setting. The *Toxoplasma* reference genome and annotations (ME49) were downloaded from ToxoDB (release 56), and data sets were mapped to the reference genome using HISAT2 (61) with default setting. The counts of reads mapping to each gene feature in the GFF annotations were completed using featureCounts (67). The outputs from featureCounts were analyzed within RStudio (Build 443) following the DESeq2 v1.32.0 vignette (68).

## Data Availability

Proteomic analysis of the TgBDP1 interactome is available at ProteomeXchange with identifier PXD038848. Sequenced reads and called peaks for TgBDP1 CUT&TAG are available as NCBI GEO GSE228853. Raw sequence reads from RNA-seq are available at NCBI Bioproject PRJNA948388.

## SUPPLEMENTAL MATERIAL

Fig. S1 mbio.03573-22-s0001.eps TgBDP1 has an mRNA isoform, TgBDP1a.

Fig. S2 mbio.03573-22-s0002.eps Parental parasite line (TATi) replicates normally in the presence of ATc.

Fig. S3 mbio.03573-22-s0003.eps Life cycle expression of genes impacted during tgbdp1 knockdown in tachyzoites.

Table S1 mbio.03573-22-s0004.xlsx Interactome analysis of TgBDP1 complex.

Table S2 mbio.03573-22-s0005.xlsx Peak calls from CUT&TAG of TgBDP1.

Table S3 mbio.03573-22-s0006.xlsx Genes with TgBDP1 binding at the TSS.

Table S4 mbio.03573-22-s0007.xlsx Genes significantly dysregulated during knockdown of tgbdp1.

Supplemental legends mbio.03573-22-s0008.docx Legends to Fig. S1 to S3 and Tables S1 to S4.

ASM does not own the copyrights to Supplemental Material that may be linked to, or accessed through, an article. The authors have granted ASM a non-exclusive, world-wide license to publish the Supplemental Material files. Please contact the corresponding author directly for reuse.
