# Supplementary material for: An apicomplexan bromodomain protein, TgBDP1, associates with diverse epigenetic factors to regulate essential transcriptional processes in Toxoplasma gondii
Source: mBio. 2023 Jun 23;14(4):e03573-22. doi: 10.1128/mbio.03573-22 (PMC10470533; doi:10.1128/mbio.03573-22)
Supplement: Supplemental legends — Legends to Fig. S1 to S3 and Tables S1 to S4. [file mbio.03573-22-s0008.docx]

**Supplemental Figure 1. TgBDP1 has an mRNA isoform, TgBDP1a.** A) Screen capture from ToxoDB genome browser showing the predicted tgbdp1 gene with exons (blue boxes) and intron (lines) on top. Underneath is predicted introns with red arrows identifying the two distinct isoforms. Nanopore sequencing read alignments of *Toxoplasma* mRNAs are shown with the 63 nucleotide isoform region flanked by red dashed lines. B) Predicted TgBDP1 protein sequence with ankyrin repeats highlighted in green, bromodomain in blue and the sequence missing from TgBDP1a in yellow. C) Multiple sequence alignment of cDNA encompassing the 63 isoform nucleotides from six different *tgbdp1* clones. Clones 1-3 have the full predicted sequence (*tgbdp1*) while clones 3-6 are missing the 63 nucleotides (*tgbdp1a*). D) Screen capture from ToxoDB genome browser showing the *tgbdp1* gene model with exons (yellow boxes) and introns (lines), with RNA-sequencing peaks of one replicate from our parasite line ^tet-myc^TgBDP1. Inset depicts the end of the first exon and beginning of the first intron, and RNA-sequencing peaks from all three replicates with the 63 nucleotide region flanked by dashed lines.

**Supplemental Figure 2. Parental parasite line (TATi) replicates normally in the presence of ATc.** A *Toxoplasma* doubling assay was performed with parasites -ATc and +ATc. The number of parasites per vacuole was counted at 12, 24 and 36hrs after inoculation.

**Supplemental Figure 3. Life cycle expression of genes impacted during *tgbdp1* knockdown in tachyzoites.** Heat maps of relative gene expression levels of the genes that are significantly downregulated (left) and upregulated (right) during *tgbdp1* knockdown. Gene expression data for entero-epithelial stages (EES) 1-5, tachyzoites and bradyzoites from Ramakrishnan *et al.* (41) were clustered according to the life cycle stage(s) in which expression peaks. Labels indicate the stage of peak gene expression for each gene cluster.

**Supplemental Table 1**

Interactome analysis of TgBDP1 complex.

**Supplemental Table 2**

Peak calls from CUT&Tag of TgBDP1.

**Supplemental Table 3**

Genes with TgBDP1 bound in proximity to the TSS.

**Supplemental Table 4**

Genes significantly dysregulated during depletion of *tgbdp1*.
